# Early Mobilization Prescription in Patients Undergoing Cardiac Surgery: Systematic Review

**DOI:** 10.21470/1678-9741-2021-0140

**Published:** 2022

**Authors:** Mayara Gabrielle Barbosa Borges, Daniel Lago Borges, Mariane Oliveira Ribeiro, Lara Susan Silva Lima, Karolina Carneiro Morais Macedo, Vinicius José da Silva Nina

**Affiliations:** 1 Department of Physiotherapy, Hospital Universitário da Universidade Federal do Maranhão (UFMA), São Luís, Maranhão, Brazil.; 2 Postgraduate Program in Health Sciences, Universidade Federal do Maranhão (UFMA), São Luís, Maranhão, Brazil.; 3 Faculty of Physiotherapy, Faculdade Pitágoras, São Luís, Maranhão, Brazil.; 4 Multiprofessional Residency Program, Hospital Universitário da Universidade Federal do Maranhão (UFMA), São Luís, Maranhão, Brazil.; 5 Department of Physiotherapy, Faculdade Pitágoras, São Luís, Maranhão, Brazil.

**Keywords:** Cardiac Surgical Procedures, Early Ambulation, Resistance Training, Intensive Care Units, Postoperative Period

## Abstract

**Introduction:**

Early mobilization of patients in the postoperative period of cardiac surgery who are hospitalized in the intensive care unit (ICU) is a practice that has a positive impact.

**Methods:**

This is a systematic review of studies published until September 2020 in the Medical Literature Analysis and Retrieval System Online (or MEDLINE®), Embase, Physiotherapy Evidence Database (or PEDro), Scientific Electronic Library Online (or SciELO), and Latin American and Caribbean Health Sciences Literature (or LILACS) databases. Randomized clinical trials describing mobilization protocols performed early in ICU patients after cardiac surgery were included.

**Results:**

According to the eligibility criteria, only 14 of the 1,128 articles found were included in the analysis. Early mobilization protocols were initiated in the immediate postoperative period or first postoperative day. The resources and technics used were progressive mobilization, cycle ergometer, early bed activities, walking protocols, resistance exercise, and virtual reality. Intensity of the mobilization activities was determined using the Borg scale and heart rate.

**Conclusion:**

Early mobilization protocols are generalist (not individual), and low-intensity exercises are used, through progressive mobilization, with two daily physical therapy sessions, during 10 to 30 minutes.

**Table:** Abbreviations, Acronyms & Symbols

6MWT	= 6-minute walk test	METs	= Metabolic equivalent of task
CABG	= Coronary artery bypass grafting	MIP	= Maximal inspiratory pressure
CPAP	= Continuous positive airway pressure	MRC	= Medical Research Council
HF	= High frequency	NR	= Not reported
HR	= Heart rate	POD	= Postoperative day
HRV	= Heart rate variability	RR	= R-R intervals
ICU	= Intensive care unit	SpO_2_	= Saturation of peripheral oxygen
IMT	= Inspiratory muscle training	VO_2_	= Oxygen uptake
LF	= Low frequency		

## INTRODUCTION

Cardiac surgery is an option to treat patients with cardiovascular disease, aiming minimize symptoms, optimize cardiac function, and increase survival. Because it is an invasive procedure, it implies numerous functional and systemic consequences in the postoperative period^[1-3]^.

Complications resulting from the surgical procedure may be caused by physiological changes, comorbidities, and previous risk factors. In addition, intraoperative conditions such as mechanical ventilation, cardiopulmonary bypass, surgical time, and anesthesia determine longer hospital stay with negative outcomes^[4]^.

Conditions acquired during hospitalization due to immobilization, such as loss of strength and muscle mass, reduction of functional capacity, and physical deconditioning, are common and directly associated with greater disability and need for prolonged rehabilitation^[5]^.

Early mobilization of patients in the postoperative period of cardiac surgery who are hospitalized in the intensive care unit (ICU) is a practice that has a positive impact on cardiovascular conditioning and ventilatory mechanics, consequently implying improvement of functional capacity, shorter hospitalization time, and lower mortality rate, besides contributing to the prevention of ICU-acquired weakness and to the improvement of muscle strength^[4,6]^.

Exercises are essential for a quick postoperative recovery. Protocols and resources to assist in cardiovascular rehabilitation have been frequently applied^[2]^. Cycle ergometer, neuromuscular electrical stimulation, virtual reality through video games, and protocols of active and resisted mobilization with levels of progression have been increasingly used, presenting positive results in functional capacity^[7,8]^ and motivation^[9]^ of patients undergoing cardiac surgery.

Thus, considering the various therapeutic proposals, this review aims to describe the prescription of early mobilization in patients undergoing cardiac surgery.

## METHODS

This is a systematic review following the recommendations of the Preferred Reporting Items for Systematic Reviews and Meta-Analyses (or PRISMA) Statement^[10]^ and registered in the International Prospective Register of Systematic Reviews (or PROSPERO) (CRD42020197787).

### Eligibility Criteria

Randomized clinical trials describing early mobilization protocols applied to patients following cardiac surgery were included. Early mobilization has been considered as any mobilization activity that has been carried out as soon as possible during the ICU stay, such as turning, sitting, and orthostatism; passive, assisted, or active exercises; marching on the spot; walking; resistance or aerobic exercise; cycle ergometer; or virtual reality games. The year of publication, as well as the language, were not considered as exclusion criteria.

### Search Strategy

The search was conducted in the Medical Literature Analysis and Retrieval System Online (or MEDLINE®) via PubMed®, Embase, Physiotherapy Evidence Database (or PEDro), Latin American and Caribbean Health Sciences Literature (or LILACS), and Scientific Electronic Library Online (or SciELO) databases.

The search strategy comprised the keywords and synonyms for “early mobilization”, and for the study population we searched for “adults undergoing cardiac surgery hospitalized in an intensive care unit”, also including interventions as “marching on the spot”, “walking”, “resistance or aerobic exercise”, “cycle ergometer”, or “virtual reality games”. The search was carried out using terms of Medical Subject Headings (or MeSH) and synonyms, without restriction of date and language, until the period of June 2020, being updated in September 2020. PubMed®'s complete search strategy is described in Table 1. Two studies were manually added after the analysis of Kanejima et al.^[11]^ study.

**Table 1:** Search strategy used in PubMed®.

#1	(“Intensive care” OR “Critical care” OR “Intensive care unit” OR “Critical illness” OR “Care, Critical” OR “Care, Intensive” OR “Surgical Intensive Care” OR “Care, Surgical Intensive” OR “Intensive Care, Surgical”)
#2	(“Early Ambulation” OR “Accelerated Ambulation” OR “Ambulation, Accelerated” OR “Ambulation, Early” OR “Early Mobilization” OR “Mobilization, Early” OR “Exercise” OR “Physical Activity” OR “Activities, Physical” OR “Activity, Physical” OR “Physical Activities” OR “Exercise, Physical” OR “Exercises, Physical” OR “Physical Exercise” OR “Physical Exercises” OR “Acute Exercise” OR “Acute Exercises” OR “Exercise, Acute” OR “Exercises, Acute” OR “Exercise, Isometric” OR “Exercises, Isometric” OR “Isometric Exercises” OR “Isometric Exercise” OR “Exercise, Aerobic” OR “Aerobic Exercise” OR “Aerobic Exercises” OR “Exercises, Aerobic” OR “Exercise Training” OR “Exercise Trainings” OR “Training, Exercise” OR “Trainings, Exercise” OR “Motion Therapy, Continuous Passive” OR “Movement Therapy, Continuous Passive” OR “Passive Movement Therapy, Continuous” OR “Continuous Passive Motion Therapy” OR “Passive Motion Therapy, Continuous” OR “Continuous Passive Movement Therapy” OR “CPM Therapy” OR “CPM Therapies” OR “Therapies, CPM” OR “Therapy, CPM” OR “Resistance Training”)
#3	(“Procedure, Cardiac Surgical” OR “Procedures, Cardiac Surgical” OR “Surgical Procedure, Cardiac” OR “Surgical Procedures, Cardiac” OR “Surgical Procedures, Heart” OR “Cardiac Surgical Procedure” OR “Heart Surgical Procedures” OR “Procedure, Heart Surgical” OR “Procedures, Heart Surgical” OR “Surgical Procedure, Heart” OR “Heart Surgical Procedure” OR “Cardiac Surgery”)
#4	#1 AND #2 AND #3 AND

Two researchers performed the initial search independently through the evaluation of titles and abstracts. Subsequently, the reviewers assessed the full texts for the independent verification of inclusion and exclusion criteria. In cases of disagreement, a third evaluator was consulted.

Data extraction was performed using a standardized Excel® spreadsheet, with the following information: first author, year of publication, country, number of patients in the study, sample, objective of the study, and, finally, description of the protocol of early mobilization (type of intervention, intensity, frequency, duration, and progression). In cases of incomplete or absent data, the corresponding authors were contacted. Data analysis was performed descriptively.

The assessment of the risk of bias in randomized controlled clinical trials followed the recommendations of the Cochrane Collaboration, using these items: random sequence generation, allocation concealment, blinding (participants, personnel, and outcome assessment), incomplete outcome data, selective reporting, and other sources of bias^[12]^.

## RESULTS

**Fig. 1:**
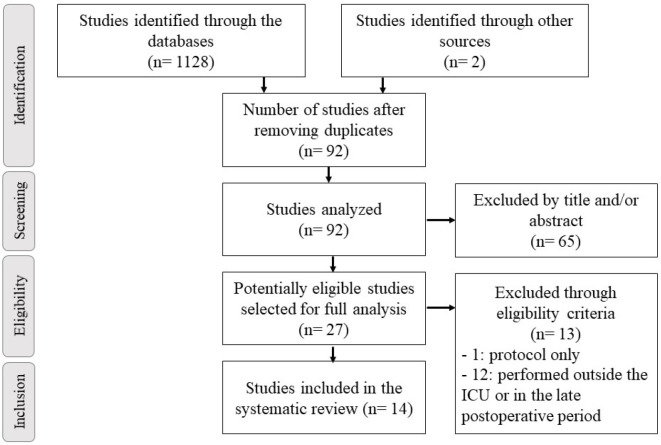
Flowchart of studies included in this systematic review. ICU=intensive care unit

The search identified 1,128 studies, but only 14 controlled and randomized clinical trials, totaling 1,170 patients included in this systematic review (Figure 1). Most studies included only coronary artery bypass grafting procedures. Mean age of patients was 58,67±4,5 years. Study characteristics are summarized in Table 2.

**Table 2:** Characteristics of the included studies.

Author	Sample	Age (years)	Sample characteristic	Objective
Borges et al.^[37]^, 2016	34	62.7±15.6	Adults undergoing CABG	To evaluate whether the addition of aerobic exercise during hospitalization improves lung function, respiratory muscle strength, and functional capacity
Cacau et al.^[7]^, 2013	60	50.6±2.5	Adults under 75 years, undergoing CABG or valve surgeries	To evaluate the use of virtual reality in functional rehabilitation
Herdy et al.^[31]^, 2008	56	59.5±9.5	Adults in the preoperative period of CABG	To evaluate the effects of cardiopulmonary rehabilitation before and after surgery in postoperative outcomes
Hirschhorn et al.^[18]^, 2007	88	62.9±8.9	Adults undergoing CABG	To assess whether a supervised walking program with or without musculoskeletal or respiratory exercises can improve walking capacity and other outcomes
Hojskov et al.^[19]^, 2019	310	65±8.8	Adults undergoing CABG	To assess the impact of phases 1 and 2 of cardiovascular rehabilitation on functionality, physical and mental function, anxiety, depression, sleep, pain, and quality of life
Gama Lordello et al.^[9]^, 2020	228	57.7±13	Adults undergoing CABG and/or valve surgeries	To evaluate the effect of early use of cycle ergometer, compared with conventional therapy, on in-hospital mobility
Mendes et al.^[35]^, 2010	47	59±8.5	Adults undergoing CABG	To determine whether a short exercise protocol during in-hospital cardiac rehabilitation can improve cardiac autonomic regulation
Pantoni et al.^[14]^, 2016	27	57.85±7.3	Adults undergoing CABG	To evaluate the effectiveness of CPAP on the first day of walking
Silva et al.^[15]^, 2017	19	52±17	Adult undergoing elective cardiac surgery (corrections of congenital heart diseases, CABG, valve surgeries, and/or associated surgical procedures)	To check the cardiorespiratory repercussions of early sitting out of bed and its effects on muscle strength, functional capacity, and pulmonary function
Stein et al.^[16]^, 2009	20	63.5 ± 6.5	Adults undergoing CABG	To evaluate the effect of a cardiopulmonary rehabilitation program on inspiratory muscle strength and its possible association with maximum and submaximal functional capacity
Tariq et al.^[17]^, 2017	174	51.9±13.8	Adults undergoing CABG or valve surgeries	To determine the effect of physical activities (≤ 3 METs) in the immediate postoperative period on respiratory and hemodynamic parameters
Ximenes et al.^[2]^, 2015	34	60.9±6.8	Adults undergoing CABG	To evaluate the effects of early resistance exercise
Windmoller et al.^[23]^, 2020	31	60±7	Adults undergoing CABG	To evaluate the effectiveness of cycle ergometer with CPAP
Zanini et al.^[22]^, 2019	40	58.5±6.25	Adults undergoing CABG	To evaluate the effect of different rehabilitation protocols on pulmonary function and functional capacity

CABG=coronary artery bypass grafting; CPAP=continuous positive airway pressure; METs=metabolic equivalent of task

Age data were expressed by mean ± standard deviation

Early mobilization protocols were initiated in the immediate postoperative period or first postoperative day. Different resources and techniques were used to mobilize patients submitted to cardiac surgery: progressive mobilization (four studies), cycle ergometer (three studies), out of bed activities (two studies), walking protocols (three studies), resistance exercise (one study), and virtual reality (one study). The description of the mobilization protocols is shown in Table 3.

**Table 3:** Early mobilization protocols in patients undergoing cardiac surgery.

Authors	Mode	Intensity	Frequency	Duration	Progression
Borges et al.^[37]^, 2016	Active and assisted exercises and progressive walking + active cycle ergometer	“As much as possible”	ICU: twice daily	1st and 2nd POD: 10 minutes	Time
			Ward: once daily	From the 3rd POD: 20 minutes	
Cacau et al.^[7]^, 2013	Metabolic exercises and mobilization using virtual reality	NR	NR	NR	Progressive METs
Herdy et al.^[31]^, 2008	Progressive exercises (passive, walking, and climbing stairs)	NR	Once daily	NR	2-4 METs
Hirschhorn et al.^[18]^, 2007	1^st^ POD: marching on the spot (3 × 1 minute) and sitting out of bed	Borg: 3 a 4/10	Twice daily	Variable, depending on the patient’s condition	Increased walking distance and time
	2^nd^ POD: assisted walking (300 meters in the morning and 5 minutes in the afternoon)				
	3^rd^ POD: assisted walking (at least 5 minutes in the morning and afternoon)				
	4^th^ POD until discharge: supervised walking with increments of 2.5 minutes, as tolerated, up to 10 minutes				
Hojskov et al.^[19]^, 2019	1^st^ to 7^th^ POD: walking, shoulder and neck mobilization, and cycle ergometer	NR	NR	NR	NR
Gama Lordello et al.^[9]^, 2020	Cycle ergometer of upper and lower limbs. After drain removal, progressive activity for orthostatism, sitting on the chair, and walking in the ICU corridor	NR	Twice daily	10 minutes	NR
Mendes et al.^[35]^, 2010	Active-assisted exercises	Exercise HR = HR rest + 20 bpm	Once daily	NR	2 to 4 METs
	STEP 1: 5 × 10 repetitions in Fowler’s position				
	STEP 2: 2 × 15 repetitions in sitting position				
	STEP 3: 3 × 15 repetitions in sitting position				
	STEP 4: 3 × 15 repetitions in sitting position + 10 minutes of walking				
	STEP 5: 3 × 15 repetitions in orthostatism + 10 minutes of walking + climbing 4 floor of stairs				
Pantoni et al.^[14]^, 2016	CPAP (10-12 cmH2O) during exercises	Exercise HR = HR rest + 20 bpm	Twice daily	NR	2 to 4 METs
	1^st^ POD: upper and lower extremity exercises				
	3^rd^ POD: active exercises and 5 minutes of walking				
	4th POD: active exercises and 10 minutes of walking				
	5th POD: 10 minutes of walking and stair training				
Silva et al.^[15]^, 2017	Sitting out of bed at 1st POD, active exercises, and progressive walking	NR	NR	30 minutes	NR
Stein et al.^[16]^, 2009	1^st^ POD: hip and knee flexion (2 × 15 repetitions), upper limbs active exercises (flexion and abduction up 90° - 2 × 10 repetitions), knees and wrist flexion and extension (3 minutes each)	NR	NR	NR	Walked distance
	2^nd^ POD: marching on the spot after mediastinal drain removal (3 × 1 to 3 minutes)				
	3^rd^ POD: walking - 100 to 200 meters				
	4^th^ POD: walking - 200 to 300 meters				
	5^th^ POD: walking - 300 to 400 meters and climbing 15 steps				
	6^th^ POD: walking - 500 to 600 meters and climbing 15 steps				
Tariq et al.^[17]^, 2017	Immediate postoperative period: sitting on the edge of bed (assisted by the physiotherapist) for 5 minutes. Orthostatism (1-2 minutes), marching on the spot (10 steps), and sitting on the chair (90 minutes)	NR	NR	NR	≤ 3 METs
Ximenes et al.^[2]^, 2015	Resistance exercises and progressive walking	Borg (does not specify value)	ICU: twice daily Ward: once daily	30 minutes	Positioning (45° on bed, sitting on the edge of bed and orthostatism)
Windmoller et al.^[23]^, 2020	Cycle ergometer with CPAP (10 cmH2O) (once daily, from 2nd to 4th POD) + exercises:	Resting HR + 30 bpm	Twice daily	20 to 30 minutes	2-6 METs
	STEP 1: supine - active exercises				
	STEP 2: sitting - active exercises				
	STEP 3: orthostatism - passive stretching of lower limbs and walking (35 to 60 meters)				
	STEP 4: orthostatism - passive stretching of lower limbs and active stretching of upper limbs, walking (60 to 100 meters), and climbing 1 floor of stairs				
	STEP 5: orthostatism - active stretching of upper and lower limbs, active exercises, walking (100 to 150 meters), and climbing up/down 1 floor of stairs				
	STEP 6: orthostatism - active stretching of upper and lower limbs, active exercises, walking (150 to 200 meters), and climbing up/down 2 floors of stairs				
	STEP 7: orthostatism - active stretching of upper and lower limbs, active exercises, walking (> 200 meters), and climbing up/down 3 floors of stairs				
Zanini et al.^[22]^,2019	Group 1: active exercises (shoulders, hips, knees, and ankle flexions), IMT, progressive walking, and conventional therapy	Borg: 11/20	Twice daily	NR	Active mobilization: series and repetition Walking: distance
	Group 2: active exercises (shoulders, hips, knees, and ankle flexions), progressive walking, and conventional therapy				
	Group 3: IMT and conventional therapy				

CPAP=continuous positive airway pressure; HR=heart rate; ICU=intensive care unit; IMT=inspiratory muscle training; METs=metabolic equivalent of task; NR=not reported; POD=postoperative day

The most frequently assessed outcomes in the studies were functional capacity, using the six-minute walk test, and respiratory muscle strength. The main results of the included studies are described in Table 4.

**Table 4:** Main outcomes of the included studies.

Author	Main outcomes
Borges et al.^[13]^, 2016	Functional capacity was maintained in the intervention group. A significant difference in functional capacity was also found in intergroup analyses at hospital discharge.
Cacau et al.^[7]^, 2013	Intervention group showed lower reduction in functional performance, decreased pain score, higher energy level, shorter hospital length of stay, and higher 6MWT distance.
Herdy et al.^[14]^, 2008	Intervention group had shorter time to endotracheal extubation, decreased incidence of pleural effusion, atelectasis, pneumonia, and atrial fibrillation or flutter, and reduced hospital length of stay.
Hirschhorn et al.^[15]^, 2007	Intervention group had significantly higher 6MWT distance at hospital discharge.
Hojskov et al.^[16]^, 2019	No significant differences between groups in 6MWT. Anxiety and depression were decreased in intervention group.
Lordello et al.^[9]^, 2020	No significant difference was found in the total number of steps between the groups. However, self-reports indicated better motivation in the intervention group.
Mendes et al.^[17]^, 2010	Intervention group presented significantly higher parasympathetic HRV values, global power, non-linear HRV indexes and mean RR. Higher values of mean HR, LF (sympathetic activity), and the LF/HF (global sympathovagal balance) were found in control group.
Pantoni et al.^[14]^, 2016	Intervention group had increased exercise time, better thoracoabdominal coordination, increased ventilation during walking, increased SpO2 values at the end of walking, and reduced dyspnea rate.
Silva et al.^[15]^, 2017	Reduction of MIP in both groups, while the maximum expiratory pressure did not reduce in the intervention group. There was no change in the MRC and decrease in spirometry values in both groups at hospital discharge.
Stein et al.^[16]^, 2009	Intervention group maintained MIP measured at 7 and 30 days postoperatively, while it was significantly reduced in the control group. 6MWT distance was higher 7 days after cardiac surgery in intervention group. VO2 peak at day 30 was also higher in the intervention group.
Tariq et al.^[17]^, 2017	In the intervention group, there was an improvement in dyspnea, respiratory rate, and oxygen saturation.
Ximenes et al.^[2]^, 2015	Intervention group maintained functional capacity at hospital discharge measured by 6MWT, while control group had a significant decrease.
Windmoller et al.^[23]^, 2020	Functional capacity decreased in both groups, without significant difference in the intervention group. ICU length of stay was lower in the intervention group. In both groups there was a decrease in maximal inspiratory and expiratory pressures, as well as in the 1-min sit-to-stand test on the fourth postoperative day compared to the preoperative period.
Zanini et al.^[22]^, 2019	The 6MWT distance on the sixth postoperative day was significantly higher in groups which included early ambulation and upper and lower limbs exercise, remaining higher at 30 days post-discharge. Peak VO2 on day 30 was also higher in in the same groups. All groups achieved similar recovery of lung function

6MWT=6-minute walk test; HF=high frequency; HR=heart rate; HRV=heart rate variability; ICU=intensive care unit; LF=low frequency; MIP=maximal inspiratory pressure; MRC=Medical Research Council; RR=R-R intervals; SpO2=saturation of peripheral oxygen; VO2=oxygen uptake

**Fig. 2:**
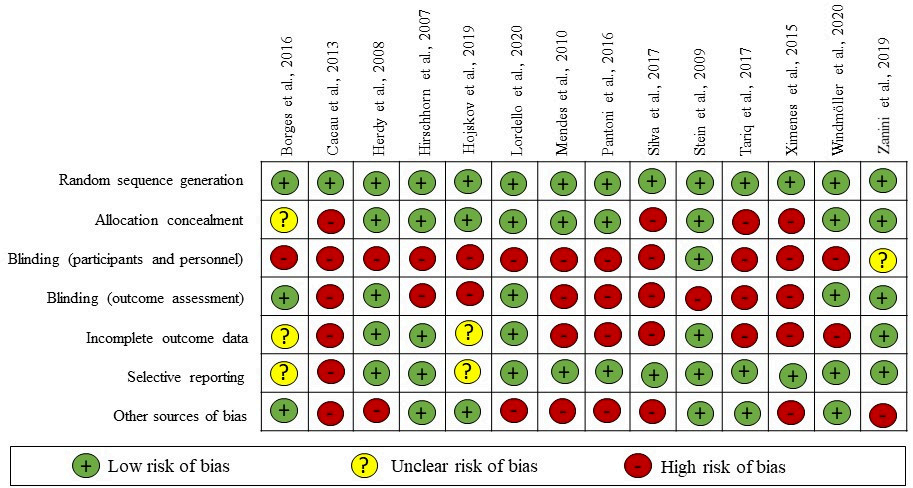
Assessment of risk of bias of the included studies.

The methodological quality of the studies was evaluated using the Cochrane tool of risk of bias, described in Figure 2. Random sequence generation, allocation concealment, and selective results have a low proportion of risk of bias. On the other hand, a high proportion of high risk of bias for blinding and other types of bias were found.

## DISCUSSION

Cardiac surgery leads to exercise capacity decreases in early stages of rehabilitation programs, when compared to patients undergoing less invasive or non-cardiac interventions^[13,14]^. Such changes are associated with severity of the disease, high prevalence of comorbidities^[15]^, duration of muscle deconditioning^[16]^, incisional pain^[17]^, chest drain, and extracorporeal circulation^[18]^.

Therefore, it is common to observe decline in functional performance during the ICU stay^[19]^. Functional capacity decrease comparing pre and postoperative periods of cardiac surgery was reported by several studies^[2,20-22]^.

Despite this, spontaneous restoration of functional capacity was observed, including in patients who did not participate in any research protocol^[23]^. In specific conditions, such as the elderly, lower training volume and longer recovery periods may be sufficient^[24]^.

Due to individual particularities, a structured therapy including mode, intensity, frequency, and duration based on individualized assessments is fundamental for a proper prescription^[25]^ and consequently long-term functional outcomes after hospital discharge^[26]^.

Early mobilization in cardiac surgery is performed in the first hours after the surgical procedure as soon as the patient presents clinical conditions for the intervention^[27]^. In the studies included in this review, the time to start mobilization was four hours after extubation to the first postoperative day.

According to Stiller et al.^[27]^ and Bourding et al.^[28]^, early mobilization after cardiac surgery promotes several benefits, including improved ventilation, ventilation/perfusion ratio, respiratory muscle strength, and functional capacity. The systematic reviews of Kanejima et al.^[11]^ and Guerra et al.^[29]^ also demonstrated positive effects on functional capacity, being considered safe and feasible in critically ill patients. On the other hand, Santos et al.^[26]^ suggests that early mobilization, evaluated in short term, does not promote significant changes in functional capacity.

Different results may be justified by divergences about early mobilization concepts^[27]^. It is important to highlight that the variety of studies with different starting points difficult the prescription, as it is essential to define the moment of initiation to avoid risks to the patient due to very early or late mobilization^[30]^.

Additionally, the term mobilization also covers several therapies. Most types of modalities found were protocols of progressive mobilization, including active exercises, sitting out of bed and walking^[31-34]^, only walking protocols^[35,36]^, and early sitting out of bed^[21,22]^. Not all these therapies require instruments for their realization.

Other studies included instruments, such as the cycle ergometer^[9,13,23]^, virtual reality^[7]^, and resistance exercises with shin pads and dumbbells^[2]^. The cycle ergometer is considered a viable strategy for those with restriction to walk^[12]^. The practice of resistance exercises in this population is restricted due to incisional precautions, but it is known that this modality optimizes cardiovascular function and peripheral muscle strength^[2,29]^ and promotes reduction of inflammation, cognitive dysfunction, and sarcopenia^[37]^.

In a systematic review, Ramos dos Santos et al.^[26]^ observed that the groups submitted to early mobilization presented lower rates of postoperative complications, improvement of functional capacity, and reduction of hospital stay in comparison with control groups without treatment. However, when comparing different mobilization protocols, there was no superiority of any intervention.

Regarding intensity, most studies^[2,7,9,13,16,19-21]^ did not use objective criteria. Hirschhorn et al.^[18]^ applied the modified Borg scale with target of three to four points, equivalent to moderate to low exercise intensity^[38]^, while Zanini et al.^[22]^ performed exercises aiming level 11 on the Borg scale from six to 20 points. This intensity corresponds to light exercises, in which participants feel that the effort is “very light”^[39]^.

In other studies, heart rate (HR) change from 20 to 30 bpm above baseline HR was used to determine exercise intensity according to guidelines from the American College of Sports Medicine (or ACSM) for patients who do not have a stress test performed. Increased exercise intensity considered the patient’s perceived effort, signs and symptoms, and normal physiological response^[40]^.

Another way to determine the exercise intensity is the reserve HR (maximum HR - resting HR)^[41]^. However, this is based on the maximum HR achieved in an effort test that can quantify the anaerobic thresholds and thus determine the prescription of adequate exercise. This type of test is not performed in early postoperative period.

Subjectively, one can also consider the speech test or Talk Test, with the perception of the ventilation itself, that is, the exercises are performed in intensity that feels the most panting breath, however, without a degree of tachypnea that prevents the patient from completing a phrase^[42]^. None of the studies analyzed used this way of determining intensity.

The definition of intensity is fundamental to determine the continuity or suspension of therapy. In phase I of the cardiac rehabilitation, low-intensity exercises should predominate, aiming the best possible physical and psychological conditions to patient hospital discharge^[43,44]^. However, it is important to highlight that the increase in exercise intensity is associated with enhanced cardiac output and oxygen consumption, resulting from increased muscle oxygen consumption. Thus, such physiological changes may be associated with greater gains in peripheral muscle strength^[45]^.

The frequency of interventions found was once to twice daily, lasting 10 to 30 minutes. The South American Guidelines for Cardiovascular Prevention and Rehabilitation^[46]^ recommended duration between 40 and 60 minutes daily. However, there is no consensus about the appropriate duration of therapy during phase I of cardiovascular rehabilitation.

Another important point in the prescription is the criteria for progression. There are protocols that demonstrate progression in steps that evolve according to patient recovery^[47,48]^ and others with progressive therapeutic strategies^[49-51]^. Winkelman et al.^[52]^ described a protocol in which each step is determined by activities with frequency and intensity corresponding to a given energy expenditure (2 to 4 metabolic equivalents of task) until hospital discharge. This form of progression was also used in several studies^[7,14,17,18,21,22]^.

The volume of therapy^[15,23]^, time^[13]^, or evolution in positioning were identified as determining factors for progression. Regardless of the form of progression, it is important to consider the functional capacity, clinical condition, use of medications, age, and objectives of the program. Moreover, in the early periods it is essential to respect the adaptation to exercise and later evolve with progression, especially in those who are reestablishing themselves from an acute event, such as cardiac surgery^[45]^.

In general, the therapy prescription is not clearly defined for patients in phase I of cardiac rehabilitation, as there is no standardization on the “dosage” of the therapy. According to the South American Guidelines for Cardiovascular Rehabilitation^[43]^, in this phase, the focus is patient education and low-intensity exercises, which include from passive mobilization to light walks with individual progressions. However, although the intensity is mild, it is important to respect the criteria of the prescription to guarantee reproducibility and efficacy of therapy, thus respecting the bases of exercise physiology.

In addition, in most of the protocols studied, it is common to perceive the same therapy for all patients. However, it is important to highlight that physical exercise, as well as drug prescription, should be individualized, aiming to maximize the benefits and minimize risks^[45]^.

Functional loss in the postoperative period is an expected complication if no therapeutic intervention is performed. So, interventions performed in this period aim to maintain functionality during the hospital stay. Divergent data were observed in the studies included in this review. Maintenance of the functional capacity in the intervention group, comparing post and preoperative periods, was found in several studies^[2,7,13,15,23]^, while others found decrease^[16,22]^ or similar values^[9]^. Concerning to respiratory function, respiratory muscle strength was decreased^[13,22]^ or maintained^[19,20]^ in both intervention and control groups, while lower incidence of respiratory complications^[14]^ was found in the intervention group . The protocols presented by Ximenes et al.^[2]^, Cacau et al.^[7]^, Borges et al.^[36]^, Stein et al.^[16]^, and Zanini et al.^[22]^ presented the best results in the most relevant outcomes.

Despite all the restrictions of the prescription of early mobilization in the postoperative period of cardiac surgery, due to the severity of the patient in phase I of cardiovascular rehabilitation, its benefits in this population are known. Thus, the question arises: would a mobilization program carefully prescribed for patients in the postoperative period of cardiac surgery be able to optimize outcomes?

## CONCLUSION

During the hospitalization phase, the prescription of early mobilization is not a frequent concern, since there are few studies specifically targeting the most appropriate type, intensity, frequency, duration, and progression. In addition, the protocols are generalist and not individual, as recommended by the physiological bases of exercise prescription. As for the studies included in the review, low-intensity exercises are used, through progressive mobilization, once to twice daily, during 10 to 30 minutes.

**Table:** Authors' roles & responsibilities

MGBB	Substantial contributions to the conception or design of the work; or the acquisition, analysis, or interpretation of data for the work; drafting the work or revising it critically for important intellectual content; final approval of the version to be published
DLB	Substantial contributions to the conception or design of the work; or the acquisition, analysis, or interpretation of data for the work; drafting the work or revising it critically for important intellectual content; final approval of the version to be published
MOR	Substantial contributions to the conception or design of the work; or the acquisition, analysis, or interpretation of data for the work; drafting the work or revising it critically for important intellectual content; final approval of the version to be published
LSSL	Substantial contributions to the conception or design of the work; or the acquisition, analysis, or interpretation of data for the work; drafting the work or revising it critically for important intellectual content; final approval of the version to be published
KCMM	Substantial contributions to the conception or design of the work; or the acquisition, analysis, or interpretation of data for the work; drafting the work or revising it critically for important intellectual content; final approval of the version to be published
VJSN	Substantial contributions to the conception or design of the work; or the acquisition, analysis, or interpretation of data for the work; drafting the work or revising it critically for important intellectual content; final approval of the version to be published
